# Influence of Low Back Pain and Prognostic Value of MRI in Sciatica Patients in Relation to Back Pain

**DOI:** 10.1371/journal.pone.0090800

**Published:** 2014-03-17

**Authors:** Abdelilah el Barzouhi, Carmen L. A. M. Vleggeert-Lankamp, Geert J. Lycklama à Nijeholt, Bas F. Van der Kallen, Wilbert B. van den Hout, Bart W. Koes, Wilco C. Peul, for the Leiden–The Hague Spine Intervention Prognostic Study Group

**Affiliations:** 1 Department of Neurosurgery, Leiden University Medical Center, Leiden, the Netherlands; 2 Department of Radiology, Medical Center Haaglanden, the Hague, the Netherlands; 3 Department of Medical Decision Making, Leiden University Medical Center, Leiden, the Netherlands; 4 Department of General Practice, Erasmus MC, University Medical Center, Rotterdam, the Netherlands; 5 Department of Neurosurgery, Medical Center Haaglanden, the Hague, the Netherlands; University of Louisville, United States of America

## Abstract

**Background:**

Patients with sciatica frequently complain about associated back pain. It is not known whether there are prognostic relevant differences in Magnetic Resonance Imaging (MRI) findings between sciatica patients with and without disabling back pain.

**Methods:**

The study population contained patients with sciatica who underwent a baseline MRI to assess eligibility for a randomized trial designed to compare the efficacy of early surgery with prolonged conservative care for sciatica. Two neuroradiologists and one neurosurgeon independently evaluated all MR images. The MRI readers were blinded to symptom status. The MRI findings were compared between sciatica patients with and without disabling back pain. The presence of disabling back pain at baseline was correlated with perceived recovery at one year.

**Results:**

Of 379 included sciatica patients, 158 (42%) had disabling back pain. Of the patients with both sciatica and disabling back pain 68% did reveal a herniated disc with nerve root compression on MRI, compared to 88% of patients with predominantly sciatica (P<0.001). The existence of disabling back pain in sciatica at baseline was negatively associated with perceived recovery at one year (Odds ratio [OR] 0.32, 95% Confidence Interval 0.18–0.56, P<0.001). Sciatica patients with disabling back pain in absence of nerve root compression on MRI at baseline reported less perceived recovery at one year compared to those with predominantly sciatica and nerve root compression on MRI (50% vs 91%, P<0.001).

**Conclusion:**

Sciatica patients with disabling low back pain reported an unfavorable outcome at one-year follow-up compared to those with predominantly sciatica. If additionally a clear herniated disc with nerve root compression on MRI was absent, the results were even worse.

## Introduction

Sciatica is associated with significant short- and sometimes long-term morbidity. This affliction, certainly in the industrialized countries, ranks as one of the most costly and ubiquitous medical problems [1]. Many synonyms for sciatica appear in the literature, such as lumbosacral radicular syndrome, ischias, radiculopathy, nerve root pain, and nerve root entrapment [2]. The literal translation of the greek word sciatica “sciatica” is hip pain, which leaves room for dispute about today's use of the word ‘sciatica’ in scientific communications [3]. Undoubtedly “lumbosacral radicular syndrome” or sciatic neuralgia is a better description of the disease but it is not frequently used in peer-reviewed articles. For this study sciatica is defined as intense leg pain in an area served by one or more spinal nerve roots and is occasionally accompanied by neurological deficit [2].

In classical literature sciatica has been of great interest to Greco-Roman and Eastern scientists and physicians [4]. For centuries an inflammation of the sciatic nerve was the origin of pain, described as sciatic neuritis [5], until 1934 when Mixter and Barr revolutionized the understanding of sciatica into mechanical origin [4], [6]. They asserted that sciatica was caused by a herniated disc pressing against a nerve root. However, soon after this landmark paper, Mixter and Ayers demonstrated in 1935 that sciatica can occur without disc herniation, arising the question whether the mechanical compression is the true origin of pain in sciatica [7]. The doubt is strengthened by several studies showing a high prevalence of disc herniations ranging from 20 to 76% in subjects without any symptoms [8], [9]. Contrary, in many people with clinical symptoms of sciatica no lumbar disc herniations are present on MRI [2], [10], [11]. Therefore it is suggested that inflammation of the nerve root may also be a major factor in sciatica and that the state of inflammation may be more important than anatomic contact between disc and nerve root [12], [13], [14].

Patients with sciatica frequently complain about associated back pain [2]. Back pain has also been reported to be associated with worse prognosis in patients with sciatica [15]. However, it remains unclear to what extent morphological changes seen on MRI in sciatica patients are associated with back pain, rather than being a representation of irrelevant differences between individuals [8], [9], [16].

The objectives of this study were to investigate MRI differences between patients who suffered both from sciatica and disabling back pain as compared to patients who suffered from sciatica only. Furthermore we report on the relevance of disabling low back pain for clinical outcome.

## Materials and Methods

### Ethics statement

The medical ethics committees at the nine participating hospitals (Leiden University Medical Center, Medical Center Haaglanden, Diaconessen Hospital, Groene Hart Hospital, Reinier de Graaf Hospital, Spaarne Hospital, Bronovo Hospital, Rijnland Hospital and Lange Land Hospital) approved the protocol. Written informed consent was obtained from all patients.

### Study population

Patients for this study were patients with intense lumbosacral nerve root pain who underwent a baseline MRI to assess the eligibility for the Sciatica Trial: a multicenter, randomized controlled trial designed to determine whether early surgery results in better outcome compared to a strategy of prolonged conservative treatment with surgery if needed among patients with 6–12 weeks sciatica [17], [18]. Patients who had symptoms being so severe that they were eligible for surgery according to their family physicians were referred to the neurologist who subsequently evaluated whether these patients were eligible to participate in the trial. Patients were excluded if they were presenting with cauda equina syndrome, severe paresis (Medical Research Counsil [MRC] <3), another episode of symptoms similar to those of the current episode during the previous 12 months, previous spine surgery, pregnancy, and severe coexisting disease. Participants who were not meeting one or more of the aforementioned exclusion criteria and had a lumbosacral radicular syndrome lasting between 6–12 weeks underwent an MRI and qualified to be included in this present study (thus for the present study it was not necessary to have a herniated disc visible on MRI). All patients with sciatica who underwent MRI (thus both randomized and non-randomized patients) were followed for one year. Patients who did not participate in the Trial were still allowed any regular treatment. Details of the design and study protocol have been published previously [18]. In the present study the data were analyzed as a cohort study (both randomized and non-randomized patients combined).

### MRI protocol and Image evaluation

MRI scans were performed in all nine participating hospitals using standardized protocols tailored to a 1.5 Tesla scanner. Sagittal T1 and axial T1 spin echo images of the lumbar spine were acquired. In addition, T2 weighted sagittal and axial series, and contrast-enhanced (gadolinium-DTPA) T1 fat suppressed images were obtained.

Two experienced neuroradiologists (BK and GL) and one neurosurgeon (CV) independently evaluated all MR images. The readers were not provided any clinical information and had not been involved in the selection or care of the included patients.

Definitions of imaging characteristics were based on recommendations from the combined task forces of the North American Spine Society, the American Society of Spine Radiology, and the American Society of Neuroradiology for classification of lumbar disc pathology [19]. Vertebral Endplate Signal Changes were defined according to criteria of Modic [20], [21]. Standardized case record forms with definitions were used to evaluate the images (Table S1 in Appendix S1).

First, the blinded readers had to decide which disc level showed the most severe nerve root compression. For both the presence of disc herniation and nerve root compression a four point scale was used: “definite about the presence”, “probable about the presence”, “possible about the presence” and “definite about the absence”. The first two categories were combined and marked as having the abnormality present. The latter two categories were combined and marked as not having the abnormality present. Clinically relevant characteristics of the disc level and disc herniation were scored. Vertebral Endplate Signal Changes were evaluated from L2–L3 through L5–S1.

### Outcomes

The patients were assessed by means of the Roland Disability Questionnaire for Sciatica (RDQ, scores range from 0 to 23, with higher scores indicating worse functional status) [22], the 100-mm visual-analogue scale (VAS) for leg and low back pain (with 0 representing no pain and 100 the worst pain ever experienced) [23], and a 7-point Likert self-rating scale of global perceived recovery with answers ranging from completely recovered to much worse. Perceived recovery was defined as “complete” or “nearly complete disappearance of symptoms” on the patient-reported 7-point Likert scale for global perceived recovery, while a score in the remaining five categories (“minimally improved”, “no change”, “minimally worse”, “much worse” and “very much worse”) was marked as “no recovery” [17], [18]. Outcome measures (both for randomized and non-randomized patients) were assessed at baseline, 2, 4, 8, 12, 26, 38 and 52 weeks.

### Statistical analysis

The majority opinion of the three readers regarding the MRI variables (answer independently given by minimum 2 out of 3 readers) was used in the statistical analysis. Interobserver agreement regarding the MRI findings was determined by use of absolute percentages of agreement and kappa values (weighted in case of ordered data).

Disabling back pain was defined as a VAS for back pain of at least 40 as this cutoff value is regularly used when the VAS is categorized into favorable and unfavorable outcome [24], [25]. Patients with missing VAS-back pain at baseline were excluded. Differences between patients with VAS-back pain of at least 40 and those with a VAS lower than 40 were assessed by using Student's t-test for continuous data and Chi-square tests for categorical data.

Logistic regression was used to determine the association between perceived recovery at one year and baseline characteristics (presence versus absence of disabling back pain at baseline, presence versus absence of disc herniation at baseline, presence versus absence of nerve root compression at baseline). The association between the baseline characteristics and perceived recovery at one year was additionally adjusted for treatment received (surgery versus no surgery), age, gender, smoking, Body Mass Index and RDQ score at baseline.

Between group differences in continuous outcome measures (RDQ and VAS pain scores) during the first year were analyzed by repeated measurement analysis of variance.

We assumed clinical outcome data to be missing at random and used model-based multiple imputation to impute the outcome values, a method in which the distribution of the observed data is used to construct sets of plausible values for the missing observations (10 imputed datasets). Variables included in the imputation model were age, gender, body-mass index, duration of symptoms, smoking, treatment group, all used MRI variables (Table S1 in Appendix S1), and baseline and other follow-up measurements of the outcomes being predicted. As sensitivity analysis we repeated the analysis using a cutoff value of 50 to define disabling back pain (disabling back pain was thus defined as a VAS for back pain of at least 50). As sensitivity analysis we also repeated the analysis using only patients with no missing data (ie no imputation). Statistical significance was defined as P<0.05.

## Results

Of the 599 patients screened for the study, 395 patients considered eligible for inclusion underwent MRI of whom 283 patients were randomized and 112 not [17], [26]. Reasons why 112 sciatica patients were not randomized was that 70 (63%) did not have a disc herniation according to the neurologist who assessed the MRI in one of the 9 participating centers at the time of enrolment (a visible disc herniation on MRI was a prerequisite to enter the Trial), 31 (28%) patients recovered before the randomization procedure could take place, and 11 (10%) patients refused to be randomized. In total, 283 baseline MRIs of the 283 randomized patients and 106 MRIs of 112 non-randomized patients could be retrieved, bringing the total to 389 MRIs. Baseline VAS of back pain was not available for 10 (2.6%) patients. Of the 379 eligible patients for the present study 139 were randomized to early surgery, 142 to prolonged conservative care and 98 not randomized. Of the 139 patients randomized to early surgery 16 recovered before surgery could be performed. Of the 142 patients in the prolonged conservative care group, 55 eventually underwent surgery within the first year. Of the 98 non-randomized patients 9 underwent surgery within the first year. Thus in total 187 patients underwent surgery.

Of the 379 patients, 158 (42%) had a VAS of at least 40 with a mean of 63.3 (95% Confidence Interval [CI] 61–66) and 221 (58%) patients had a VAS of back pain of less than 40 with a mean VAS of 12.1 (95% CI 11–14). At baseline, sciatica patients with and without disabling back pain had a statistically significant but clinically small difference in RDQ and VAS-leg pain (17.4 vs. 15.0 and 66.6 vs. 60.7 respectively) (Table 1). Six of the 379 patients (1.6%) had bilateral nerve root pain. Clinical outcome at 52 weeks was missing in 12–13% of patients (Table S2 in Appendix S1). Baseline RDQ and VAS for leg and back pain were comparable among patients for whom clinical outcome at 52 weeks was available and those for whom not (P-value range 0.21–0.42).

**Table 1:** Baseline characteristics stratified by presence of disabling back pain.

Variable	Sciatica with disabling back pain (n = 158)	Sciatica with no disabling back pain (n = 221)	P-value
Age at baseline MRI	42.8±10.9	43.4±9.6	0.56
Male-sex	92 (58)	147 (67)	0.09
Duration of sciatica (weeks)	9.0±2.4	9.5±3.8	0.11
BMI||	26.1±4.2	25.9±3.6	0.59
Treatment group			0.09
Non-randomized	48 (30)	50 (23)	
Randomized to early surgery	60 (38)	79 (36)	
Randomized to prolonged conservative care	50 (32)	92 (42)	
Smoking	67 (42)	80 (36)	0.24
Roland disability score for sciatica¶			
Baseline	17.4±3.3	15.0±4.5	<0.001
12 months	4.5±5.9	2.9±4.7	0.004
Visual-analogue scale of leg pain‡			
Baseline	66.6±20.3	60.7±22.7	0.009
12 months	13.7±22.4	8.7±16.5	0.014
Visual-analogue scale of back pain‡			
Baseline	63.3±16.2	12.1±11.6	<0.001
12 months	21.3±26.1	12.2±18.8	<0.001
Perceived recovery∫			
12 months	111 (70)	195 (88)	<0.001

Values are n (%) or means ± SD.

|| Body-mass index (BMI) is the weight in kilograms divided by the square of the height in meters.

¶ The Roland disability questionnaire for sciatica is a disease-specific disability scale that measures functional status in patients with pain in the leg or back. Scores range from 0 to 23, with higher scores indicating worse functional status.

‡ The intensity of pain was indicated on a horizontal 100 mm visual analogue scale, with 0 representing no pain and 100 the worst pain ever experienced.

∫ Perceived recovery was defined as complete or nearly complete disappearance of symptoms according to the Likert-7 point scale.

Substantial agreement was found for the MRI assessed presence of disc herniation (kappa range 0.67–0.75) and nerve root compression (kappa range 0.60–0.80) (Table S3 in Appendix S1). Moderate agreement was found for the size of the disc herniation (kappa range 0.35–0.55) and presence of vertebral endplate signal changes (kappa range 0.49–0.67).

### MRI differences with and without disabling back pain

Of patients with both sciatica and disabling back pain 76% had a disc herniation on MRI compared to 91% of patients without disabling back pain (P<0.001) (Table 2). Nerve root compression on MRI was observed less frequently in patients with both disabling sciatica and back pain compared to patients with predominantly sciatica (68% vs. 88%, P<0.001). No significant differences existed in prevalence of Vertebral Endplate Signal Changes between sciatica patients with and without disabling back pain (41% vs. 43%, P = 0.70).

**Table 2:** Comparison of MRI characteristics between sciatica patients with and without disabling back pain at baseline.

	Sciatica with disabling back pain (n = 158)	Sciatica with no disabling back pain (n = 221)	P-value
*MRI characteristic*			
Presence of disc herniation	120 (76)	202 (91)	**<0.001**
Presence of nerve root compression	108 (68)	195 (88)	**<0.001**
Presence of Vertebral Endplate Signal Changes at one or more lumbar level¶	63 (41)	94 (43)	0.91
Type 1	3 (5)	6 (6)	
Type 2	58 (92)	84 (89)	
Type 3	0 (0)	1 (1)	
Mixed Type 1 and 2	2 (3)	3 (3)	
Presence of Schmorl's nodules (herniation of the disc into the vertebral-body endplate) at one or more levels	18 (12)	25 (11)	0.94

Values are n (%).

¶ Vertebral Endplate Signal Changes were defined according to criteria of Modic and their presence was assessed from vertebral endplates L2–L3 through L5–S1. Type 1 lesions: hypointense in T1-weighted sequences and hyperintense in T2-weighted sequences. Type 2 lesions: increased signal on T1 weighted sequences and isointense or slightly hyperintense signal on T2 weighted sequences. Type 3 lesions: hypointense both in T1- and T2-weighted sequences.

A comparison of the characteristics of the herniated disc itself between sciatica patients with and without disabling back pain is shown in Table 3. Large disc herniations (size >50% of spinal canal) were observed in an equal percentage (18%) between patients with and without disabling back pain. Also, no significant difference existed in extrusions between patients with and without disabling back pain (64% vs. 67%, P = 0.66).

**Table 3:** Comparison of the characteristics of the herniated disc on MRI between sciatica patients with and without disabling back pain at baseline.

	Sciatica with disabling back pain (n = 125)	Sciatica with no disabling back pain (n = 205)	P-value
Size of disc herniation			
Size >50% in relation to spinal canal	23 (18)	37 (18)	0.95
Size <50% in relation to spinal canal	102 (82)	167 (81)	
Not classifiable	0 (0)	1 (1)	
Location of disc herniation			
Central and/or subarticular	111 (89)	183 (89)	0.70
Foraminal and/or extraforaminal	14 (11)	20 (10)	
Not classifiable	0 (0)	2 (1)	
Morphology of disc herniation			
Extrusion	80 (64)	138 (67)	0.66
Protrusion	42 (34)	65 (32)	
Not classifiable	3 (2)	2 (1)	
Loss of disc height at the disc level of the disc herniation			
Yes	112 (90)	186 (91)	0.96
No	10 (8)	17 (8)	
Not classifiable	3 (2)	2 (1)	
Signal intensity of nucleus pulposus on T2 images at the disc level of the disc herniation			
Hypointensity	110 (88)	185 (90)	0.72
Normal	10 (8)	15 (7)	
Hyperintensity	(0)	1 (1)	
Not classifiable	5 (4)	4 (2)	
Presence of Vertebral Endplate Signal Changes at the disc level of the disc herniation¶			
Type 1	2 (4)	6 (7)	0.70
Type 2	51 (93)	76 (91)	
Type 3	0 (0)	0 (0)	
Mixed Type 1 and 2	2 (4)	2 (2)	

N = 330. Values are n (%).

¶ Vertebral Endplate Signal Changes were defined according to criteria of Modic. Type 1 lesions: hypointense in T1-weighted sequences and hyperintense in T2-weighted sequences. Type 2 lesions: increased signal on T1 weighted sequences and isointense or slightly hyperintense signal on T2 weighted sequences. Type 3 lesions: hypointense both in T1- and T2-weighted sequences.

### Clinical outcome in relation to disabling back pain and MRI differences

The existence of disabling back pain in sciatica at baseline was negatively associated with perceived recovery at one year (Odds ratio [OR] 0.32, 95% CI 0.18–0.56, P<0.001) (Table 4). This result was consistent with the continuous outcomes RDQ and VAS pain scores (Figure S1 in Appendix S1). By contrast, presence of disc herniation on MRI at baseline was positively associated with perceived recovery at one year (OR 3.18, 95% CI 1.58–6.39, P = 0.001) (Table 4). The presence of nerve root compression on MRI at baseline was also positively associated with perceived recovery at one year (OR 4.99, 95% CI 2.70–9.24, P<0.001).

**Table 4:** Perceived recovery at one year according to presence of disabling back pain and the presence of disc herniation or nerve root compression on MRI at baseline.

	UnivariateAnalysis OR (95% CI)	P-value	Adjusted for received treatment OR (95% CI)¶	P-value	Multivariate adjustmentOR (95% CI)‡	P-value
Presence of disabling back pain at baseline	0.32 (0.18–0.56)	<0.001	0.31 (0.17–0.56)	<0.001	0.34 (0.17–0.67)	0.002
Presence of disc herniation on MRI	3.18 (1.58–6.39)	0.001	3.04 (1.37–6.72)	0.006	3.16 (1.28–7.81)	0.01
Presence of nerve root compression on MRI	4.99 (2.70–9.24)	<0.001	4.91 (2.50–9.64)	<0.001	5.54 (2.62–11.75)	<0.001

OR denotes odds ratio. CI denotes confidence interval. Total n = 379.

Perceived recovery was defined as “complete” or “nearly complete disappearance of symptoms” on the 7-point Likert scale.

¶ Analysis adjusted for actual treatment received (surgery vs. no surgery during the first year).

‡ Analysis adjusted for actual treatment received (surgery vs. no surgery during the first year), age, gender, body-mass index, smoking and Roland Disability Questionnaire score at baseline.

The reported prevalence of perceived recovery at one year was 81% for sciatica patients who had at baseline disabling back pain and nerve root compression, 50% for sciatica patients who had at baseline back pain but no nerve root compression, 91% for sciatica patients who had at baseline no back pain but depicted nerve root compression on MRI, and 73% for sciatica patients who had at baseline no back pain and no nerve root compression (P<0.001) (Table 5). In the stratified analysis according to treatment group the overall trends were comparable with the non-stratified analysis (Table S4 in Appendix S1).

**Table 5:** Clinical outcome measures at one year according to subgroups at baseline.

	Clinical outcome at one year			
	Perceived recovery∫	Roland Disability‡	VAS-Leg pain¶	VAS-back pain¶
**Subgroups according to back pain and presence of nerve root compression on MRI at baseline**				
Back pain and nerve root compression (n = 108)	87 (81)	3.6±5.8	11.8±21.7	17.4±23.9
Back pain and no nerve root compression (n = 50)	25 (50)	6.4±5.8	17.8±23.5	29.6±28.8
No back pain and nerve root compression (n = 195)	177 (91)	2.7±4.4	7.6±14.1	11.4±17.2
No back pain and no nerve root compression (n = 26)	19 (73)	4.5±6.6	16.7±27.9	18.7±27.4
**Subgroups according to back pain and presence of disc herniation on MRI at baseline**				
Back pain and disc herniation (n = 120)	90 (75)	4.2±6.2	14.4±23.9	20.0±26.2
Back pain and no disc herniation (n = 38)	22 (58)	5.4±5.1	11.6±16.8	25.2±25.8
No back pain and disc herniation (n = 202)	181 (90)	2.8±4.5	7.7±14.1	11.6±17.3
No back pain and no disc herniation (n = 19)	14 (74)	4.1±6.5	18.8±31.7	18.3±29.9

Subgroups defined by the presence of disabling back pain and the presence of a disc herniation or nerve root compression on MRI at baseline. Values are n (%) or means ± SD. N = 379.

∫ Perceived recovery was defined as complete or nearly complete disappearance of symptoms according to the Likert-7 point scale.

‡ The Roland Disability Questionnaire for Sciatica is a disease-specific disability scale that measures the functional status of patients with pain in the leg or back. Scores range from 0 to 23, with higher scores indicating worse functional status.

¶ The intensity of pain is indicated on a horizontal 100 mm visual analogue scale (VAS) with 0 representing no pain and 100 the worst pain ever experienced.

**Figure 1:**
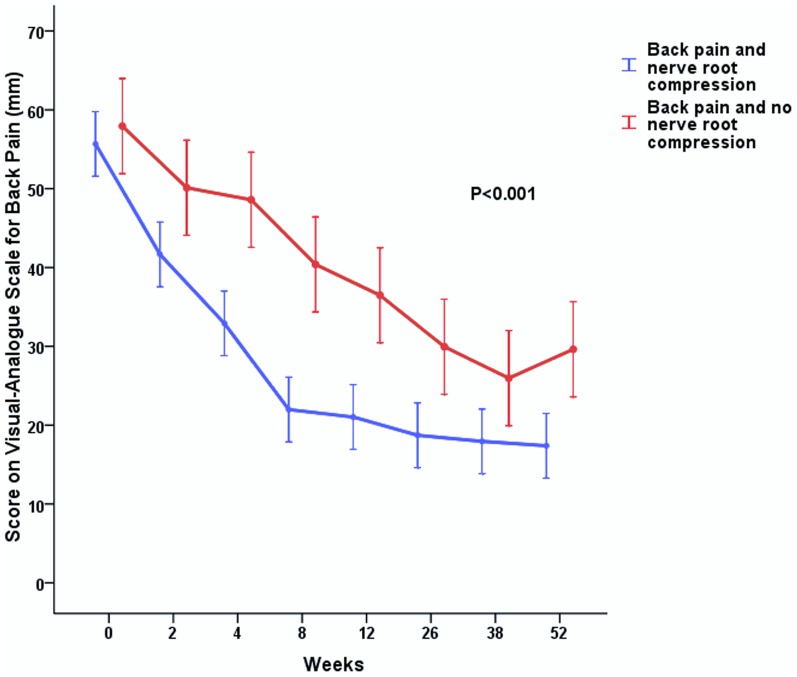
Repeated measurement analysis curve of Mean Scores for back pain on the Visual-Analogue Scale. Sciatica patients with both disabling back pain and nerve root compression on MRI were compared with patients with disabling back pain but who did not depict nerve root compression on MRI at baseline. The vertical bars represent 95% confidence intervals.

In patients with disabling back pain, those who also had nerve root compression on MRI significantly reported more favorable recovery from their back pain at one year compared to those who had not depicted nerve root compression at baseline (Figure 1).

The sensitivity analyses yielded comparable results (Tables S5, S6 and S7 in Appendix S1).

## Discussion

In this study of patients with sciatica who were followed for one year, those with disabling back pain at baseline reported an unfavorable outcome at one-year follow-up compared to those with predominantly sciatica. If additionally a herniated disc with nerve root compression on MRI was absent, the results were even worse. Herniated discs and nerve root compression on MRI were more prevalent among patients with predominantly sciatica compared to those who suffered from additional disabling back pain. However, vertebral endplate signal changes were equally distributed between those with and without disabling back pain. Remarkably large disc herniations and extruded disc herniations were also equally distributed between the two groups.

Over the past two decades there has been an ongoing scientific debate about the clinical relevance of MRI morphological variations [8], [9]. To uncover the relevance of imaging findings, knowledge regarding their prevalence and relation with symptoms in different subgroups is needed. However, in most clinical studies, patients with herniated discs have been reported as a single pathological group [27]. Comparable to the present study, some researchers have attempted to identify MRI differences between subgroups. MRI differences have been reported between patients with both sciatica and low back pain compared to asymptomatic control subjects [8], and between sciatica patients compared to low back pain patients [10]. The finding in the present study that vertebral endplate signal changes were equally distributed between those with and without disabling back pain was surprising as they are hypothesized to be a causative factor in low back pain [28], [29]. The finding in the present study that extruded disc herniations and large disc herniations were also equally distributed between the two groups was also surprising as both findings have been reported to correlate with the severity of symptoms in sciatica [8], [16]. However, these studies [8], [16] did not compare these findings between sciatica patients with and without back pain. Comparable to the present study, Vroomen described a more favourable prognosis for patients with compared to those without nerve root compression on MRI [30].

The preoccupation with the herniated disc as a source of disabling low back and leg pain has led disc surgery to become one of the most commonly performed operative procedures. However, disc herniations are often seen on imaging studies in patients without symptoms [8], [9]. Contrary, in the present study, a substantial number of patients without disc herniation or nerve compression suffered from sciatica. The worldwide accepted mechanical compression theory therefore seems not to offer a sufficient explanation for the cause of the disabling back and leg symptoms in sciatica. Some researchers suggested that nerve root inflammation may also be a major factor in sciatica [12], [13], [14]. Further research is needed to reveal the pain mechanisms in sciatica and how therapeutic strategies should be applied accordingly.

The results after lumbar disc surgery do not seem to have improved during recent decades. Depending upon the used outcome measure, both classical studies and recent randomized controlled trials show that during longer follow-up treatment results for sciatica are satisfactory in 60 to 85% of the patients [17], [26], [31], [32], [33]. The number of proposed interventions, developed by numerous disciplines, is overwhelming. The results of this study indicate that in sciatica subgroups with different prognostic profiles can be identified. A shift from a “one-size fits all” approach, where heterogeneous groups of patients receive broadly similar treatments, towards targeted treatments according to prognostic profiles or specific characteristics, may help to improve the treatment results [34].

A strength of this study was the blinded MRI assessment and follow-up of all patients with 6–12 weeks sciatica who underwent MRI, regardless of participation in the randomized trial. A limitation of the present study is that the study population consisted of sciatica patients who had severe symptoms and were referred to the neurologists. These patients were willing to undergo surgery, so patients with a clear preference for conservative treatment are underrepresented. Some might view the agreement among MRI readers as suboptimal. However, the kappa values are comparable with those found in previous studies [9], [35], [36] and therefore one might consider them to reflect existing agreement among expert readers in clinical practice. Finally, some researchers might have chosen the Oswestry Disability Questionnaire (ODQ) as an outcome instead of the RDQ. At the design phase of this study it was known that the ODQ might be better when evaluating patients with persistent severe disability [37]. However, as sciatica generally has a favorable prognosis we anticipated that most of the patients would report less disability at follow-up visits. At lower levels of disability it was known that RDQ scores may discriminate better than ODQ scores [37]. One study also had found fewer incomplete or ambiguous responses to the RDQ than to the Oswestry questionnaire [38]. Another study observed that responsiveness of the RDQ was better than of the ODQ scale [39].

## Conclusions

Sciatica patients with disabling low back pain reported an unfavorable outcome at one-year follow-up compared to those with predominantly sciatica. If additionally a clear herniated disc with nerve root compression on MRI was absent, the results were even worse. Further research is needed to identify the reasons behind the different prognostic profiles in sciatica and how to apply new or existing therapeutic strategies accordingly.

## Supporting Information

Appendix S1 Contains list of authors and participants in the Leiden–The Hague Spine Intervention Prognostic Study Group and Tables S1–S7, Figure S1. **Table S1**, MRI study variables. **Table S2**, Outcome measurements available at 52 weeks after baseline MRI. **Table S3**, Interobserver agreement regarding the MRI characteristics. **Table S4**, Clinical outcome measures at one year stratified according to subgroups at baseline and treatment group. **Table S5**, Perceived recovery at one year according to presence of disabling back pain at baseline. **Table S6**, Clinical outcome measures at one year according to subgroups at baseline. **Table S7**, Clinical outcome measures at one year according to subgroups at baseline. **Figure S1**, Repeated measurement analysis curves of Mean Scores on the Roland Disability Questionnaire (1A), the Visual-Analogue Scale for leg pain (1B), and the Visual-Analogue Scale for back pain (1C) in relation to disabling back pain at baseline.
(DOC)
