# Supplementary material for: Influence of Low Back Pain and Prognostic Value of MRI in Sciatica Patients in Relation to Back Pain
Source: PLoS One. 2014 Mar 17;9(3):e90800. doi: 10.1371/journal.pone.0090800 (PMC3956604; doi:10.1371/journal.pone.0090800)
Supplement: Appendix S1 — Contains list of authors and participants in the Leiden–The Hague Spine Intervention Prognostic Study Group and Tables S1–S7, Figure S1. Table S1, MRI study variables. Table S2, Outcome measurements available at 52 weeks after baseline MRI. Table S3, Interobserver agreement regarding the MRI characteristics. Table S4, Clinical outcome measures at one year stratified according to subgroups at baseline and treatment group. Table S5, Perceived recovery at one year according to presence of disabling back pain at baseline. Table S6, Clinical outcome measures at one year according to subgroups at baseline. Table S7, Clinical outcome measures at one year according to subgroups at baseline. Figure S1, Repeated measurement analysis curves of Mean Scores on the Roland Disability Questionnaire (1A), the Visual-Analogue Scale for leg pain (1B), and the Visual-Analogue Scale for back pain (1C) in relation to disabling back pain at baseline. (DOC) [file pone.0090800.s001.doc]

**Appendix S1**

**Authors and participants in the Leiden–The Hague Spine Intervention Prognostic Study Group and their role in the currently presented study were as follows:**

*Conception and design of the clinical part of the study:* B.W. Koes, W.B. van den Hout, R.T.W.M. Thomeer, and W.C. Peul

*Conception and design of the diagnostic part (with regard to the value of MRI) of the study:* A. el Barzouhi, W.B. van den Hout, and W.C. Peul

*Data Collection and Management was done by the following research nurses:* M. Nuyten, P. Bergman, G. Holtkamp, S. Dukker, A. Mast, L. Smakman, C. Waanders, L. Polak, and A. Nieborg

*MRI blinded reading:* C.L.A.M. Vleggeert-Lankamp, G.J. Lycklama à Nijeholt, B.F. Van der Kallen

*Statistical Analysis of the data*: A. el Barzouhi and W.B. van den Hout

*Interpretation of the data:* A. el Barzouhi, C.L.A.M. Vleggeert-Lankamp, G.J. Lycklama à Nijeholt, B.F. Van der Kallen, WB van den Hout, B.W. Koes, and W.C. Peul

*Vouch for the data and analysis*: A. el Barzouhi, C.L.A.M. Vleggeert-Lankamp, G.J. Lycklama à Nijeholt, B.F. Van der Kallen, WB van den Hout, B.W. Koes, and W.C. Peul

*Manuscript Preparation (first draft):* A. el Barzouhi

*Critical revision for important intellectual content:* C.L.A.M. Vleggeert-Lankamp, G.J. Lycklama à Nijeholt, B.F. Van der Kallen, WB van den Hout, B.W. Koes, and W.C. Peul

*Decision to publish the paper:* A. el Barzouhi, C.L.A.M. Vleggeert-Lankamp, G.J. Lycklama à Nijeholt, B.F. Van der Kallen, WB van den Hout, B.W. Koes, and W.C. Peul

*Sponsors of the study*: a grant from the Netherlands Organisation for Health Research and Development (ZonMW) and the Hoelen Foundation, The Hague.

*Role of the sponsor:* there were no agreements concerning confidentiality of the data between the sponsor and the authors or the institutions named in the article or in this supplement. The sponsors did also not have any role in the writing or analysis of this study.

*Protocol Committee:* W.C. Peul, B.W. Koes, and R.T.W.M. Thomeer

*Steering Committee:*  B.W. Koes, R.T.W.M. Thomeer, J.A.H. Eekhof, J.T.J. Tans, W.B. van den Hout, W.C. Peul, R. Brand, and H.C. van Houwelingen

*Participating Hospitals and Coordinating Physicians:* Medical Center Haaglanden, The Hague — J.T.J. Tans and R. Walchenbach; Diaconessen Hospital, Leiden — J. van Rossum, P. Schutte, and R.T.W.M. Thomeer; Groene Hart Hospital, Gouda — G.A.M. Verheul, J.E. Dalman, and J.A.L. Wurzer; Reinier de Graaf Hospital, Delft/Voorburg — J.W.A. Sven and A. Kloet; Spaarne Hospital, Heemstede/Haarlem — I.S.J. Merkies and H. van Dulken; Bronovo Hospital, The Hague — P.C.L.A. Lambrechts and J.A.L. Wurzer; Haga Hospital, The Hague — R.W.M. Keunen and C.F.E. Hoffmann; Rijnland Hospital, Leiderdorp/Alphen ad Rijn — J. Haan and H. van Dulken; Lange Land Hospital, Zoetermeer — R. Groen and R.R.F. Kuiters; Leiden University Medical Center, Leiden — R.A.C. Roos and J.H.C. Voormolen; Public Health and Primary Care, Leiden University, Leiden — J.A.H. Eekhof.

**Table S1** **MRI study variables.** The three readers (2 neuroradiologists and one neurosurgeon) independently used the same case record form.

| **MRI variable** | **Type** | **Categories** |
| --- | --- | --- |
| Disc level with the most severe nerve root compression | Disc level | 1. L2L3  2. L3L4  3. L4L5  4. L5S1  5. Not applicable, all disc levels have a normal disc contour: no disc extension beyond the normal margins of the intervertebral disc space at any disc level |
|  | Disc contour at this disc level | 1. Bulging: presence of disc tissue circumferentially (50-100%) beyond the edges of the ring apophyses  2. Herniation: localized displacement of disc material beyond the normal margins of the intervertebral disc space  3. Not applicable, all disc levels have a normal disc contour: no disc extension beyond the normal margins of the intervertebral disc space at any disc level |
|  | Certainty about the presence of this disc herniation | 1. Definite about the presence: no doubt about the presence  2. Probable about the presence: some doubt but probability > 50%  3. Possible about the presence: reason to consider but probability < 50%  4. Definite about the absence: no doubt about the absence |
|  | Loss of disc height (distance between the planes of the end-plates of the vertebrae craniad and caudad to the disc) at this disc level | 1. Yes  2. No |
|  | Signal intensity of nucleus pulposus on T2 images at this level | 1. Hypointensity  2. Normal  3. Hyperintensity |
| If a herniation at the disc level is considered | Side of this disc herniation | 1. Right  2. Left  3. Right and left |
|  | Location on axial view of this disc herniation | 1. Central zone: zone within the vertebral canal between sagittal planes through the medial edges of each facet  2. Sub-articular zone: zone, within the vertebral canal, sagittally between the plane of the medial edges of the pedicles and the plane of the medial edges of the facets, and coronally between the planes of the posterior surfaces of the vertebral bodies and the under anterior surfaces of the superior facets.  3. Foraminal zone: zone between planes passing through the medial and lateral edges of the pedicles  4. Extra-foraminal zone: the zone beyond the sagittal plane of the lateral edges of the pedicles, having no well-defined lateral border. |
|  | Size of this disc herniation in relation to spinal canal | 1. Large stenosing: size >75% of the spinal canal  2. Large: size 75-50% of the spinal canal  3. Average: size 25-50% of the spinal canal  4. Small: size <25% of the spinal canal |
|  | Morphology | 1. Protrusion: localized displacement of disc material beyond the intervertebral disc space, with the base against the disc of origin broader than any other dimension of the protrusion.  2. Extrusion: localized displacement of disc material beyond the intervertebral disc space, with the base agains the disc of origin narrower than any one distance between the edges of the disc material beyond the disc space measured in the same plane, or when no continuity exists between the disc material beyond the disc space and that within the disc space. |
| Nerve root compression | Certainty about the presence of nerve root compression | 1. Definite about the presence: no doubt about the presence  2. Probable about the presence: some doubt but probability > 50%  3. Possible about the presence: reason to consider but probability < 50%  4. Definite about the absence: no doubt about the absence |
| Separate for every end plate from level L2-L3 through L5-S1 | Presence of vertebral endplate signal changes (VESC) | 1. No VESC  2. VESC type 1: hypointense in T1-weighted sequences and hyperintense on T2-weighted sequences  3. VESC type 2: increased signal on T1 weighted sequences and isointense or slightly hyperintense signal on T2 weighted sequences  4. VESC type 3: hypointense both on T1- and T2-weighted sequences  5. VESC type 1 and 2 |
|  | Presence of Schmorl’s nodes (herniation of the disc into the vertebral-body end plate) | 1. Yes  2. No |

**Table** **S2 Outcome measurements available at 52 weeks after baseline MRI.** The mentioned outcome measures were assessed at baseline, 2, 4, 8, 12, 26, 38, and 52 weeks. Values are n (%). Total n=379

|  | Number of patients (%) |
| --- | --- |
| **Visual Analogue scale for back pain at 52 weeks**¶ |  |
| Outcome available at 52 weeks | 332 (88) |
| At least one follow-up examination | 37 (10) |
| Lost to follow-up after baseline examination | 10 (3) |
| **Global perceived recovery on a 7-point Likert scale at 52 weeks**∫ |  |
| Outcome available at 52 weeks | 330 (87) |
| At least one follow-up examination | 39 (10) |
| Lost to follow-up after baseline examination | 10 (3) |
| **Roland disability questionnaire at 52 weeks**‡ |  |
| Outcome available at 52 weeks | 333 (88) |
| At least one follow-up examination | 36 (9) |
| Lost to follow-up after baseline examination | 10 (3) |
| **Visual Analogue scale for leg pain at 52 weeks**¶ |  |
| Outcome available at 52 weeks | 334 (88) |
| At least one follow-up examination | 35 (9) |
| Lost to follow-up after baseline examination | 10 (3) |

¶ The intensity of pain is indicated on a horizontal 100 mm visual analogue scale (VAS) with 0 representing no pain and 100 the worst pain ever experienced.

∫ Global perceived recovery was defined as complete or nearly complete disappearance of symptoms according to the Likert-7 point scale.

‡ The Roland Disability Questionnaire for Sciatica is a disease-specific disability scale

that measures the functional status of patients with pain in the leg or back. Scores range from 0 to 23, with higher scores indicating worse functional status.

**Table S3 Interobserver agreement regarding the MRI characteristics.** Reader A en B represent the two neuroradiologists, while reader C represents the neurosurgeon. Kappa values and percentages of agreement for the characteristics of disc herniation were only calculated if the observers agreed about their presence (e.g. when a reading pair showed disagreement about the presence of disc herniation, this patient did not contribute to the interagreement analysis regarding the characteristics of the herniated disc).

|  | A vs B | | A vs C | | B vs C | | All observers | |
| --- | --- | --- | --- | --- | --- | --- | --- | --- |
|  | %  agreement | kappa | %  agreement | kappa | %  agreement | kappa | %  agreement | multirater  kappa |
| Disc level with the most severe nerve root compression ¶ | 92.0 | 0.86 | 88.4 | 0.81 | 90.5 | 0.84 | 86.4 | 0.84 |
| Probability of disc herniation (2 categories) ‡ | 93.6 | 0.75 | 91.8 | 0.71 | 90.0 | 0.67 | 87.7 | 0.71 |
| Probability of nerve root compression (2 categories) ‡ | 94.1 | 0.80 | 85.4 | 0.62 | 84.6 | 0.60 | 82.0 | 0.66 |
| Presence of vertebral end plate changes ∫ | 73.8 | 0.49 | 83.4 | 0.67 | 81.0 | 0.60 | 69.1 | 0.58 |
| Presence of Schmorl’s nodes ∫ | 80.3 | 0.25 | 81.6 | 0.47 | 82.6 | 0.26 | 72.2 | 0.33 |
| Characteristics disc herniation |  |  |  |  |  |  |  |  |
| Location axial view ╞ | 94.2 | 0.88 | 95.5 | 0.90 | 96.7 | 0.93 | 95.6 | 0.92 |
| Size disc herniation in relation to spinal canal  (2 categories) ║ | 82.1 | 0.55 | 76.3 | 0.35 | 86.3 | 0.47 | 71.5 | 0.44 |
| Protrusion versus extrusion | 77.4 | 0.48 | 75.0 | 0.50 | 73.7 | 0.44 | 63.2 | 0.46 |
| Loss of disc height of the disc level ∫ | 97.9 | 0.86 | 72.2 | 0.26 | 72.4 | 0.26 | 71.5 | 0.31 |
| Signal intensity of nucleus pulposus on T2 images § | 95.3 | 0.75 | 90.4 | 0.64 | 90.7 | 0.57 | 88.6 | 0.61 |

¶ The 5 categories were: 1) L2L3 2) L3L4 3) L4L5 4) L5S1 5) Not applicable, all disc levels have a normal disc contour: no disc extension beyond the normal margins of the intervertebral disc space at any disc level.

‡ The categories "Definite and probable about the presence" were combined to one category and the categories “possible about the presence” and “definite about the absence” were also combined to one category.

∫ Categories were: yes versus no.

╞ Categories were: 1) Central zone 2) Sub-articular zone 3) Foraminal zone 4) Extra-foraminal zone.

║ The categories "large stenosing" and “large” were combined to one category and the categories “average” and “small” were also combined to one category.

§ Categories were: 1) Hypointensity 2) Normal 3) Hyperintensity.

**Table S4 Clinical outcome measures at one year stratified according to subgroups at baseline and treatment group.** Values are n (%) or means ± SD.

|  | Clinical outcome at one year | | | |
| --- | --- | --- | --- | --- |
|  | Perceived recovery∫ | Roland Disability‡ | VAS-Leg pain¶ | VAS-back pain¶ |
| **Patient not randomized** |  |  |  |  |
| Back pain and nerve root compression (n=14) | 11 (79) | 3.4±4.2 | 5.9±8.5 | 18.5±21.3 |
| Back pain and no nerve root compression (n=34) | 16 (47) | 6.3±5.2 | 14.8±17.3 | 29.2±2.6 |
| No back pain and nerve root compression (n=33) | 31 (94) | 2.7±3.9 | 7.3±13.6 | 7.5±13.0 |
| No back pain and no nerve root compression (n=17) | 11 (65) | 5.7±7.1 | 20.6±32.3 | 22.8±31.2 |
| **Patients assigned to surgery** |  |  |  |  |
| Back pain and nerve root compression (n=52) | 42 (81) | 3.4±6.2 | 12.9±22.2 | 15.6±22.2 |
| Back pain and no nerve root compression (n=8) | 4 (50) | 8.7±7.4 | 34.7±33.6 | 36.6±34.4 |
| No back pain and nerve root compression (n=73) | 69 (95) | 2.4±4.4 | 6.7±14.5 | 10.9±18.6 |
| No back pain and no nerve root compression (n=6) | 4 (67) | 4.3±7.0 | 11.7±18.0 | 15.3±20.7 |
| **Patients assigned to conservative care** |  |  |  |  |
| Back pain and nerve root compression (n=42) | 34 (81) | 4.1±5.9 | 13.0±23.7 | 19.5±26.9 |
| Back pain and no nerve root compression (n=8) | 2 (25) | 9.8±5.0 | 31.8±25.4 | 39.2±32.0 |
| No back pain and nerve root compression (n=89) | 77 (87) | 3.1±4.5 | 9.1±14.0 | 13.1±17.2 |
| No back pain and no nerve root compression (n=3) | 3 (100) | 1.0±1.7 | 2.3±2.3 | 3.7±4.0 |

∫ Perceived recovery was defined as complete or nearly complete disappearance of symptoms according to the Likert-7 point scale.

‡ The Roland Disability Questionnaire for Sciatica is a disease-specific disability scale

that measures the functional status of patients with pain in the leg or back. Scores range from 0 to 23, with higher scores indicating worse functional status.

¶ The intensity of pain is indicated on a horizontal 100 mm visual analogue scale (VAS) with 0 representing no pain and 100 the worst pain ever experienced.

**Table S5 Perceived recovery at one year according to presence of disabling back pain at baseline.** Back pain was defined as a VAS (visual analogue scale) for back pain of at least 50.

|  | Univariate Analysis  OR  (95% CI) | P-value | Adjusted for received treatment  OR  (95% CI) ¶ | P-value | Multivariate adjustment  OR  (95% CI) ‡ | P-value |
| --- | --- | --- | --- | --- | --- | --- |
| Presence of disabling back pain at baseline | 0.26  (0.15-0.47) | <0.001 | 0.24  (0.13-0.44) | <0.001 | 0.24  (0.12-0.49) | <0.001 |

OR denotes odds ratio. CI denotes confidence interval.

Perceived recovery was defined as “complete” or “nearly complete disappearance of symptoms” on the 7-point Likert scale.

¶ Analysis adjusted for actual treatment received (surgery vs. no surgery during the first year).

‡ Analysis adjusted for actual treatment received (surgery vs. no surgery during the first year),

age, gender, body-mass index, smoking and Roland Disability Questionnaire score at baseline .

**Table S6 Clinical outcome measures at one year according to subgroups at baseline.** Subgroups defined by the presence of back pain *(defined as a VAS for back pain of at least 50)* and disc herniation or nerve root compression on MRI at baseline. Values are n (%) or means ± SD. N=379

|  | **Clinical outcome at one year** | | | |
| --- | --- | --- | --- | --- |
|  | Perceived recovery∫ | Roland Disability‡ | VAS-Leg pain¶ | VAS-back pain¶ |
| **Subgroups according to back pain and presence of nerve root compression on MRI at baseline** |  |  |  |  |
| Back pain and nerve root compression (n=78) | 58 (74) | 4.3±6.3 | 13.2±22.6 | 19.2±25.3 |
| Back pain and no nerve root compression (n=45) | 23 (51) | 6.3±5.8 | 17.5±23.4 | 29.7±29.2 |
| No back pain and nerve root compression (n=225) | 206 (92) | 2.6±4.3 | 7.7±14.8 | 11.5±17.5 |
| No back pain and no nerve root compression (n=31) | 20 (65) | 5.0±6.5 | 17.3±27.4 | 20.4±27.2 |
| **Subgroups according to back pain and presence of disc herniation on MRI at baseline** |  |  |  |  |
| Back pain and disc herniation (n=89) | 61 (69) | 5.0±6.6 | 16.2±25.1 | 22.3±27.8 |
| Back pain and no disc herniation (n=34) | 20 (59) | 5.1±5.0 | 11.1±15.2 | 24.9±25.6 |
| No back pain and disc herniation (n=233) | 210 (90) | 2.7±4.5 | 7.9±14.8 | 11.9±17.6 |
| No back pain and no disc herniation (n=23) | 15 (65) | 4.7±6.6 | 18.3±30.8 | 19.9±29.7 |

∫ Perceived recovery was defined as complete or nearly complete disappearance of symptoms according to the Likert-7 point scale.

‡ The Roland Disability Questionnaire for Sciatica is a disease-specific disability scale

that measures the functional status of patients with pain in the leg or back. Scores range from 0 to 23, with higher scores indicating worse functional status.

¶ The intensity of pain is indicated on a horizontal 100 mm visual analogue scale (VAS) with 0 representing no pain and 100 the worst pain ever experienced.

**Table S7 Clinical outcome measures at one year according to subgroups at baseline.** Subgroups defined by the presence of back pain *(defined as a VAS for back pain of at least 40)* and disc herniation or nerve root compression on MRI at baseline. *This analysis only included patients with available clinical outcome at one year.* Values are n (%) or means ± SD. N=330

|  | **Clinical outcome at one year** | | | |
| --- | --- | --- | --- | --- |
|  | Perceived recovery∫ | Roland Disability‡ | VAS-Leg pain¶ | VAS-back pain¶ |
| **Subgroups according to back pain and presence of nerve root compression on MRI at baseline** |  |  |  |  |
| Back pain and nerve root compression (n=101) | 80 (79) | 3.8±5.9 | 12.1±22.0 | 17.9±24.4 |
| Back pain and no nerve root compression (n=30) | 10 (33) | 8.3±5.8 | 22.8±25.6 | 35.9±30.5 |
| No back pain and nerve root compression (n=176) | 161 (91) | 2.6±4.4 | 7.1±13.1 | 10.9±16.5 |
| No back pain and no nerve root compression (n=23) | 16 (70) | 4.7±6.9 | 17.9±28.8 | 19.7±28.3 |
| **Subgroups according to back pain and presence of disc herniation on MRI at baseline** |  |  |  |  |
| Back pain and disc herniation (n=111) | 82 (74) | 4.4±6.3 | 14.9±24.4 | 20.8±26.8 |
| Back pain and no disc herniation (n=20) | 8 (40) | 6.9±5.2 | 12.9±15.6 | 28.3±27.1 |
| No back pain and disc herniation (n=185) | 167 (90) | 2.8±4.5 | 7.3±13.2 | 11.3±16.7 |
| No back pain and no disc herniation (n=14) | 10 (71) | 4.1±7.0 | 22.3±34.8 | 21.1±33.2 |

∫ Perceived recovery was defined as complete or nearly complete disappearance of symptoms according to the Likert-7 point scale.

‡ The Roland Disability Questionnaire for Sciatica is a disease-specific disability scale

that measures the functional status of patients with pain in the leg or back. Scores range from 0 to 23, with higher scores indicating worse functional status.

¶ The intensity of pain is indicated on a horizontal 100 mm visual analogue scale (VAS) with 0 representing no pain and 100 the worst pain ever experienced.

**Figure S1 Repeated measurement analysis curves of Mean Scores on the Roland Disability Questionnaire (1A), the Visual-Analogue Scale for leg pain (1B), and the Visual-Analogue Scale for back pain (1C) in relation to disabling back pain at baseline.** The vertical bars represent 95% confidence intervals.

**S1A** Curve for the mean Roland Disability Questionnaire (scores range from 0 to 23, with higher scores indicating worse functional status) in relation to disabling back pain at baseline.

**
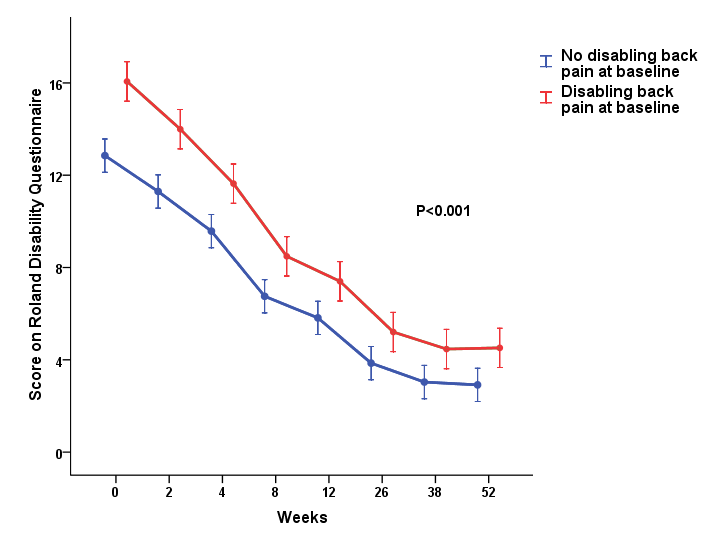
**

**S1C** Curve for the mean scores on the visual-analogue scale for intensity of leg pain (scale ranges from 0 to 100 mm, with higher scores indicating more intense pain) in relation to disabling back pain at baseline.

**
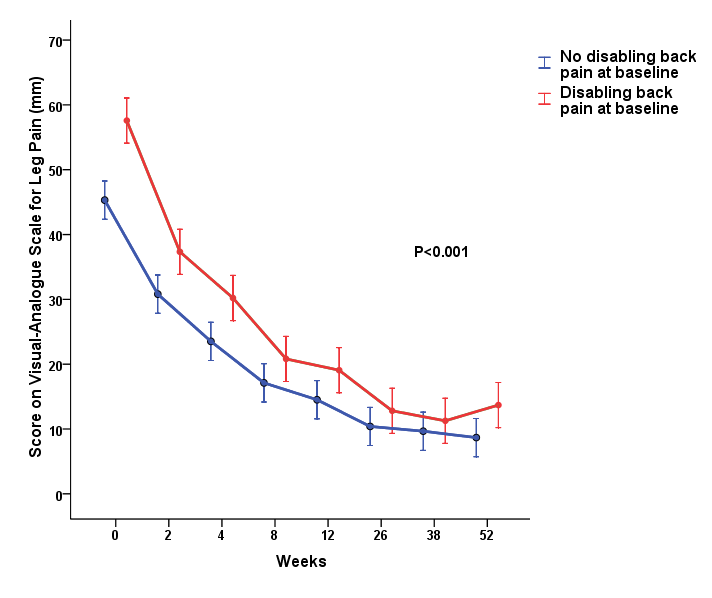
**

**S1C** Curve for the mean scores on the visual-analogue scale for intensity of back pain (scale ranges from 0 to 100 mm, with higher scores indicating more intense pain) in relation to disabling back pain at baseline.

**
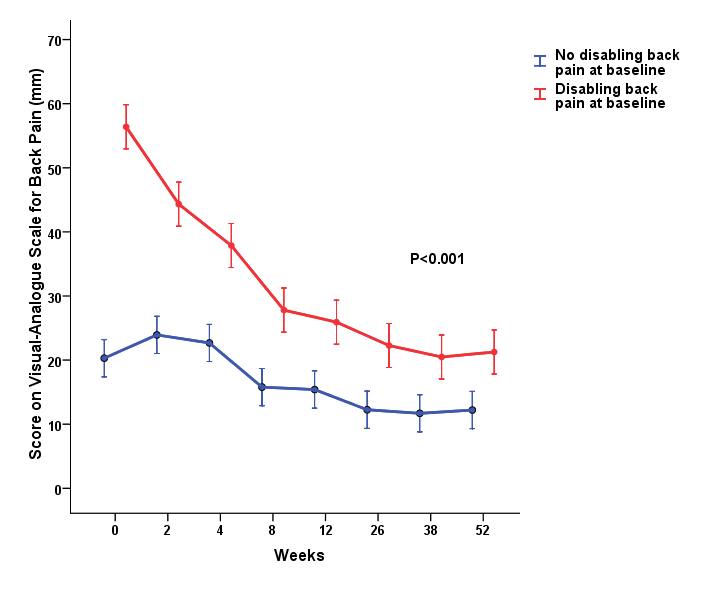
**
